# Supplementary material for: Bayesian refinement of protein structures and ensembles against SAXS data using molecular dynamics
Source: PLoS Comput Biol. 2017 Oct 18;13(10):e1005800. doi: 10.1371/journal.pcbi.1005800 (PMC5662244; doi:10.1371/journal.pcbi.1005800)
Supplement: S1 Archive — (BZ2) [file pcbi.1005800.s011.tar.bz2 › gromacs_4.62_bayesian_saxs_refi/share/html/online/g_dist.html]

g\_dist


| g\_dist Main Table of Contents |

**VERSION 4.6  
Sat 19 Jan 2013**

---

### Description

g\_dist can calculate the distance between the centers of mass of two
groups of atoms as a function of time. The total distance and its
x-, y-, and z-components are plotted.

Or when -dist is set, print all the atoms in group 2 that are
closer than a certain distance to the center of mass of group 1.

With options -lt and -dist the number of contacts
of all atoms in group 2 that are closer than a certain distance
to the center of mass of group 1 are plotted as a function of the time
that the contact was continuously present. The -intra switch enables
calculations of intramolecular distances avoiding distance calculation to its
periodic images. For a proper function, the molecule in the input trajectory
should be whole (e.g. by preprocessing with trjconv -pbc) or a matching
topology should be provided. The -intra switch will only give
meaningful results for intramolecular and not intermolecular distances.

Other programs that calculate distances are g\_mindist
and g\_bond.

### Files

| option | filename | type | description |
| --- | --- | --- | --- |
| **-f** | traj.xtc | Input | Trajectory: xtc trr trj gro g96 pdb cpt |
| **-s** | topol.tpr | Input | Run input file: tpr tpb tpa |
| **-n** | index.ndx | Input, Opt. | Index file |
| **-o** | dist.xvg | Output, Opt. | xvgr/xmgr file |
| **-lt** | lifetime.xvg | Output, Opt. | xvgr/xmgr file |

### Other options

| option | type | default | description |
| --- | --- | --- | --- |
| **-[no]h** | bool | no | Print help info and quit || **-[no]version** | bool | no | Print version info and quit || **-nice** | int | 19 | Set the nicelevel || **-b** | time | 0 | First frame (ps) to read from trajectory || **-e** | time | 0 | Last frame (ps) to read from trajectory || **-dt** | time | 0 | Only use frame when t MOD dt = first time (ps) || **-xvg** | enum | xmgrace | xvg plot formatting: xmgrace, xmgr or none || **-[no]intra** | bool | no | Calculate distances without considering periodic boundaries, e.g. intramolecular. || **-dist** | real | 0 | Print all atoms in group 2 closer than dist to the center of mass of group 1 |

---

http://www.gromacs.org  
gromacs@gromacs.org
